# Psychometric evaluation of a pragmatic measure of clinical supervision as an implementation strategy

**DOI:** 10.1186/s43058-023-00419-1

**Published:** 2023-04-06

**Authors:** Mimi Choy-Brown, Nathaniel J. Williams, Nallely Ramirez, Susan Esp

**Affiliations:** 1 University of Minnesota, Twin Cities, 1404 Gortner Avenue, St. Paul, MN 55108 USA; 2 Boise State University, 1910 University Drive, Education Suite 717, Boise, ID 83725-1940 USA

**Keywords:** Evidence-based practice, Implementation, Clinical supervision, Measure development

## Abstract

**Background:**

Valid and reliable measurement of implementation strategies is essential to advancing implementation science; however, this area lags behind the measurement of implementation outcomes and determinants. Clinical supervision is a promising and highly feasible implementation strategy in behavioral healthcare for which pragmatic measures are lacking. This research aimed to develop and psychometrically evaluate a pragmatic measure of clinical supervision conceptualized in terms of two broadly applicable, discrete clinical supervision techniques shown to improve providers’ implementation of evidence-based psychosocial interventions—(1) audit and feedback and (2) active learning.

**Methods:**

Items were generated based on a systematic review of the literature and administered to a sample of 154 outpatient mental health clinicians serving youth and 181 community-based mental health providers serving adults. Scores were evaluated for evidence of reliability, structural validity, construct-related validity, and measurement invariance across the two samples.

**Results:**

In sample 1, confirmatory factor analysis (CFA) supported the hypothesized two-factor structure of scores on the Evidence-Based Clinical Supervision Strategies (EBCSS) scale (*χ*^2^=5.89, *df*=4, *p*=0.208; RMSEA=0.055, CFI=0.988, SRMR=0.033). In sample 2, CFA replicated the EBCSS factor structure and provided discriminant validity evidence relative to an established supervisory alliance measure (*χ*^2^=36.12, *df*=30, *p*=0.204; RMSEA=0.034; CFI=0.990; SRMR=0.031). Construct-related validity evidence was provided by theoretically concordant associations between EBCSS subscale scores and agency climate for evidence-based practice implementation in sample 1 (*d*= .47 and .55) as well as measures of the supervision process in sample 2. Multiple group CFA supported the configural, metric, and partial scalar invariance of scores on the EBCSS across the two samples.

**Conclusions:**

Scores on the EBCSS provide a valid basis for inferences regarding the extent to which behavioral health providers experience audit and feedback and active learning as part of their clinical supervision in both clinic- and community-based behavioral health settings.

**Trial registration:**

ClinicalTrials.gov NCT04096274. Registered on 19 September 2019.

**Supplementary Information:**

The online version contains supplementary material available at 10.1186/s43058-023-00419-1.

Contributions to the literature

- Measurement of implementation strategies lags behind other implementation constructs. Limited accurate and practical measurement stalls efforts to make evidence-informed decisions about effective methods to promote the implementation of programs and practices.
- This study advances the conceptualization of clinical supervision as an implementation strategy and provides evidence for the validity of a pragmatic measure of evidence-based clinical supervision strategies.
- This study helps fill a measurement gap in implementation science by providing a tool for implementation researchers and practitioners to evaluate and optimize embedded clinical supervision techniques as a lever to promote routine integration of evidence-based practices.

## Background

Sound measurement is foundational to implementation science, and while many authors have noted the need for improved measurement of implementation outcomes and determinants [1], far less attention has been paid to the measurement of implementation strategies, which arguably represent the heart of the field [2]. Implementation strategies are the methods used to change healthcare practice; they represent the means through which patient or provider behavior is modified to improve the use of evidence-based treatments [3]. Much attention has been devoted to operationalizing [2, 4, 5] and categorizing [6–8] implementation strategies, often with the explicit goal of facilitating their precise measurement [2]. However, despite these advances, the development of *measures* of implementation strategies has lagged far behind other areas [1, 9]. This measurement deficit has stalled efforts to assess the use of implementation strategies in community settings—for the purpose of identifying areas of strength and targets for improvement [2]—and has hindered the consolidation of research findings on the effects of implementation strategies across studies [10]. This paper describes the development and psychometric evaluation of a measure of one implementation strategy—clinical supervision—which is highly feasible for acting on numerous implementation outcomes across stages of implementation in settings where behavioral healthcare is delivered.

### Operationalizing clinical supervision as an implementation strategy

Clinical supervision is included within taxonomies of implementation strategies, which define it broadly as “provid[ing] clinicians with ongoing supervision focusing on the innovation” and “provid[ing] training for clinical supervisors who will supervise clinicians who provide the innovation” [6]. While these definitions are useful for distinguishing the overarching process of clinical supervision from other implementation strategies, such as expert consultation, we propose that precise measurement of clinical supervision as an implementation strategy benefits from a more granular conceptualization of the specific techniques used within supervision time to facilitate practice change [11]. Delineation and measurement of techniques used by supervisors to facilitate specific implementation outcomes will enable greater clarity regarding exactly *what* facilitates implementation outcomes and will enhance harmonization of scientific findings across studies. Thus, we propose that the measurement of clinical supervision as an implementation strategy should focus on discrete supervision techniques that (a) occur within broader supervision interactions and (b) have the highest potential for impact on implementation outcomes within community behavioral healthcare.

Research on clinical supervision has identified two discrete techniques which are associated with improved implementation outcomes and are applicable across psychosocial behavioral health interventions: [1] audit and feedback and [2] active learning [12–15]. Both of these techniques include behaviors that could occur outside of supervision; however, both fit naturally within the supervision process and have long been considered important elements of effective clinical supervision [16–18]. A recent systematic review [11] confirmed that these two supervision techniques, long considered “gold standard” components of supervision by researchers [12], are associated with improved implementation of clinical practices in behavioral health settings. Given the importance of pragmatism in implementation measurement [19], and the possibility that these techniques may represent a “minimum intervention necessary for change” [20], we propose that the assessment of these two techniques within the context of clinical supervision represents a valuable starting point for operationalizing and measuring clinical supervision as an implementation strategy.

### Gaps in measuring clinical supervision as an implementation strategy

Guidelines for the development of implementation measures stress the importance of optimization with regard to three criteria—reliability, validity, and pragmatism [19, 21]. No available measures of clinical supervision strategies are optimal on all three criteria [22]. Coder-rated observational measures, such as the Supervision Process Observational Coding System [12], can be considered gold-standard measures with strong evidence of reliability and validity [12, 23]; however, the requirements of coding audio-recorded sessions using trained raters (a rare practice outside of training clinics or clinical trials) significantly limits their pragmatism [24, 25]. Measures that rely on clinician or supervisor report are more feasible [26–29]; however, available measures are either too narrow, focusing in great depth on only a single clinical intervention, or too broad, assessing only the duration, format, and general functions of supervision (e.g., crisis assessment) rather than the use of specific supervision techniques that facilitate implementation across clinical interventions. Furthermore, many measures lack strong evidence of score reliability or validity. In sum, the field lacks measures of clinical supervision that have strong evidence of validity and that meet criteria for pragmatism including free, brief, easy to administer, and understandably written [30]. This is a significant barrier to the widespread evaluation of clinical supervision as an implementation strategy in both routine care and research trials.

### Study aims

The aim of this research was to develop and evaluate a reliable, valid, and pragmatic measure of clinical supervision, conceptualized as an overarching implementation strategy comprised of two, evidence-based and broadly applicable techniques: [1] audit and feedback and [2] active learning. In aim 1, investigators developed items for the Evidence-Based Clinical Supervision Strategies (EBCSS) scale and evaluated evidence of score reliability, structural validity, and construct-related validity in a sample of clinicians delivering outpatient psychotherapy to youth and their families. In aim 2, the items were administered to a sample of providers delivering community-based mental health services to adults and evidence of score validity was assessed with regard to measures of theoretically important supervision constructs. In aim 3, investigators tested the extent to which scores on the EBCSS exhibited measurement invariance across the two samples from aims 1 and 2.

## Methods

### Item generation

Items were generated for the EBCSS within two domains of [1] audit and feedback and [2] active learning. Audit and feedback was defined as the review and use of information regarding a supervisee’s clinical performance to identify ways to optimize the delivery of new programs or practices [6]. Three types of clinical performance information could be incorporated into the audit and feedback process: symptom monitoring, which involves examining data from client outcome measures; review of practice, which involves the supervisor’s observation of therapeutic interactions between the practitioner and the client (either in person, via audio or video recordings, or through documentation); and fidelity assessment, which involves examining data about the practitioner’s use of an evidence-based treatment as intended by the developers [31]. A recent systematic review and meta-analysis concluded that the effects of audit and feedback were strongest when feedback was delivered by supervisors as compared to other sources [32]. Providing feedback informed by clinical performance information has been key to improving the competent delivery of care [25, 33] and is successfully used as an implementation strategy in nearly every supervision outcomes study to support high-fidelity delivery of evidence-based practices (EBP) [11, 34]. On their own, neither observation (audit) nor feedback is sufficient to promote the implementation and sustainment of new clinical intervention; consequently, they were conceptualized and measured as an integrated unit.

Active learning was defined as using behavioral strategies to solidify the application of concepts into practice [16, 35]. According to experiential learning theory, skills and knowledge are acquired through a process of practical experience, reflection, conceptualization, and planning [36]. Clinical supervision provides a holding environment for this learning process, grounded in practice experience and contextual adaptation, and facilitated by the supervisor-supervisee relationship [37]. Using active learning strategies, such as behavioral rehearsal (also referred to as role play), in supervision sessions has been associated with improved adoption and fidelity to EBP in subsequent treatment sessions with clients [13, 38]. In addition, behavioral rehearsal within supervision is a pragmatic and valid method for evaluating clinicians’ fidelity [35, 39].

After generating definitions of each domain based on the literature, the research team reviewed existing supervision measures, including observational measures (e.g., SPOCS)[12], for potentially relevant item stems and content [40–43]. Items were then drafted to elicit supervisee reports of their supervision experience during the prior 30-day period. The research team and two consulting clinical supervisors reviewed and revised items iteratively until a consensus was reached on item content and wording. For the audit and feedback domain, items included three primary sources of clinical performance feedback: symptom ratings, observation of practice, and documentation. Items for the active learning domain included both behavioral rehearsal and supervisor modeling of skills.

### Participants and procedures

The aim 1 sample included clinicians who participated in a baseline survey of a larger study aimed at understanding how to support the implementation of EBPs in mental health settings serving youth. Outpatient mental health clinics were eligible to participate if they provided psychotherapy to youth and their families and were located in one of three western States in the USA targeted for enrollment. Clinicians working in these agencies were eligible to participate if they delivered psychotherapy to youth on a 50% or greater full-time equivalent basis.

Participating clinicians in this sample received an email invitation from the research team to complete a confidential web-based survey in October and November of 2019. Participants provided electronic informed consent prior to responding and received a $30 gift card. In total, *N*=21 agencies, employing *N* =193 eligible clinicians participated in the study; *N*=177 clinicians responded to the survey representing a response rate of 92%. The final analytic sample included *N*=154 clinicians who indicated they participated in clinical supervision. To evaluate the statistical power associated with this sample size, we used guidelines and Monte Carlo simulation code provided by Wolf et al. [44]. Assuming a two-factor confirmatory factor analysis (CFA) model with the hypothesized factor structure, small to moderate factor loadings of 0.65, and a moderate factor correlation of 0.50 (based on the anticipated correlation of the two supervision techniques), *N*=140 participants were adequate to generate 0.9 statistical power for all parameters of interest [45]. All procedures were approved by the affiliated Institutional Review Board.

The aim 2 sample included providers working on Assertive Community Treatment (ACT) teams in two States in the USA. ACT is an interdisciplinary team-based model providing community-based health services for adults diagnosed with a severe mental illness [46]. All assertive community treatment team leaders (*N*=52*)* working in these two states received an electronic invitation to enroll their teams in the survey and 77% (*N*=40) of the teams were enrolled. Providers (*N*=181) working on an enrolled team responded to an email invitation to participate in the web-based survey from May to July 2021, representing an average provider response rate of 50%. Participants were asked to provide electronic informed consent prior to participation and received a $20 electronic gift card. All procedures were approved by the affiliated Institutional Review Board. Based on simulation procedures described by Wolf et al. [44], we determined that a sample size of *N*=180 was adequate to achieve power >0.8 for all parameters of interest in aim 2, assuming the hypothesized CFA factor structure, medium factor loadings of 0.65 [45], and small to moderate factor correlations ranging from 0.40 to 0.55, based on the anticipated relationship between the EBCSS subscales and the measure of supervisory alliance.

The aim 3 sample was comprised of the samples from aims 1 and 2. Simulation research by Sass and colleagues [47] indicates our total sample of *N*=335 participants provides adequate statistical power (>0.8) to test our measurement invariance hypotheses given our data (i.e., ordinal categorical indicators), model specification, and choice of estimator.

The STROBE checklist of items to include in reports of observational studies was used for this study (see Additional File 2).

### Measures

The extent to which supervisees experienced *audit and feedback* and *active learning* in their clinical supervision during the last 30 days was assessed using the five EBCSS items developed for this project as described above. Each item included a statement describing a specific supervision experience and clinicians indicated how often it occurred during the last 30 days, using a 5-point Likert-type scale from 1 (“*Never*”) to 5 (“*Almost Always*”). Coefficient alpha for both subscales were acceptable in both samples (i.e., α > 0.7).

In addition, participants reported on general supervision characteristics including total hours of supervision time in a typical week; percentage of supervision time typically focused on clinical content (e.g., case conceptualization, treatment interventions), administrative content (e.g., billing), or “other” content (e.g., professional development); and perceptions of their supervisor’s availability when they have a question, ranging from 1 (“almost never”) to 5 (“almost always”).

In addition to the measures described above, clinicians in sample 1 rated their agency’s *EBP implementation climate* using the 18-item Implementation Climate Scale (ICS) [48]. The ICS assesses the extent to which clinicians share perceptions that they are expected, supported, and rewarded to use EBP in their clinical work with clients. Scores on the ICS have demonstrated excellent reliability and evidence of construct-related validity [49–52], including positive associations with EBP-related content in clinical supervision [28]. Items were rated on a Likert-type scale from 0 (“not at all”) to 4 (“a very great extent”). Coefficient alpha was 0.93 in this sample. In accordance with theory and prior research, clinician responses to the ICS were aggregated to the agency level for analysis following an assessment of interrater agreement among clinicians within each agency using the *r*_*wg(j)*_ index with a null distribution [53]. In this sample, all values of *r*_*wg(j)*_ were above the recommended cutoff of 0.7 (*M* = 0.92, *SD* = 0.07), supporting the use of the agency-level aggregate scores [54].

Providers in sample 2 completed four measures of their supervision experience in addition to the EBCSS items. The quality of supervisees’ *working alliance* with their supervisors was assessed using the five-item Brief Supervisory Working Alliance Inventory - Trainee Form (BSWAI-T) [55]. Providers indicated the frequency with which each item characterized their work with their supervisor along two dimensions: rapport and client focus. Items are scored on a Likert-type scale from 1 (“Almost never”) to 7 (“Almost always”). Prior research offers strong evidence supporting the reliability and validity of scores on the BSWAI-T [55]. Cronbach’s alpha in this sample was *α* = 0.81.

The *quality of the supervisory exchange* between supervisees and their supervisors was assessed using the 7-item Leader-Member Exchange [56]. The scale was generated to capture the quality of supervisor-supervisee interactions [57]. An example item is: “How would you characterize your working relationship with your leader?” Scores on the scale range from 7 (very low-quality exchanges) to 35 (high-quality exchanges). Decades of prior research has established the psychometric validity and utility of this measure for characterizing supervisory process and relationships [58] and it has been used in mental health treatment settings [59]. Coefficient alpha was excellent in this sample (*α* = 0.92).

The extent to which supervisors engaged in leadership behaviors that supported ACT implementation (*ACT leadership*) was assessed using 11 items generated from a study in which ACT experts rated the importance of specific supervisor behaviors for supporting high adherence to the ACT model [45]. Behaviors included in this scale were rated as extremely important by experts (> 6 on a 1 to 7 scale) and addressed four domains, including facilitating team meetings, enhancing provider skills, monitoring outcomes, and quality improvement. Coefficient alpha was excellent in this sample (*α* = 0.95).

The extent to which supervisees experienced *inadequate supervision behaviors* in their supervision was assessed using ten items from the harmful and inadequate supervision scale [60]. This scale is grounded in theory and expert ratings of supervisory behaviors that may insufficiently support supervisees and has been tested in the USA and Ireland [60, 61]. Seven items were selected for this study from the “inadequate” supervision behaviors subscale, representing global experiences of supervision (e.g., supervision is a waste of time, supervisee provided consent or a contract for supervision) that were consistent with supervision models in mental health [62] and not redundant with other items in the study. In addition, three items were generated for this study that focused specifically on attention to racism and power in supervision (e.g., supervisor interest in staff experiences of racism in their work). Coefficient alpha was good in this sample (*α* = 0.85).

### Data analysis

For aim 1, internal consistency reliability of the EBCSS subscale scores was evaluated using Cronbach’s alpha (SPSS version 27). Confirmatory factor analysis (CFA) was used to assess structural validity evidence. Given the hypothesized two-factor structure, a correlated 2-factor model was specified, with items assessing active learning forced to load on one factor and items assessing audit and feedback forced to load on another factor. Models were estimated in Mplus 8.0 using robust maximum likelihood estimation (MLR) which is appropriate for nonnormally distributed variables and small samples [63–66]. Model fit was evaluated using the model chi-square test, root mean square error of approximation (RMSEA), comparative fit index (CFI), and standardized root mean square residual (SRMR) [67]. A non-significant model chi-square test supports the hypothesized model by failing to reject it [68]. Commonly accepted thresholds of RMSEA are <0.05 for close fit, <0.08 for reasonable fit, and >0.10 indicating poor fit [67, 68]. Values of CFI ≥ 0.95 and values of SRMR ≤0.05 indicate good model fit (Schreiber et al., 2006). To further test the hypothesized factor structure, an alternative 1-factor model was estimated to evaluate if responses to items were caused by a single latent construct.

Construct-related validity evidence for sample 1 was generated by using two-level linear mixed effects regression models to test the hypothesis that scores on the EBCSS would be higher in agencies with higher levels of EBP implementation climate. These models incorporated random agency intercepts [69, 70] and were implemented in Mplus [66] using the TYPE=TWOLEVEL command and default MLR estimator. Clinician years of experience and level of education (doctoral vs. non-doctoral) were included as covariates to isolate the association of climate with the EBCSS subscales. Because agency climate should only influence supervisors who work within an agency, the sample for this analysis was restricted to clinicians who reported receiving agency-based supervision (*N*=147). Missing data (fewer than 2% of cases) were addressed using Bayesian multiple imputation (*N*=10 datasets). Effect sizes were calculated using an analogue to Cohen’s *d* [69]. Values represent the standardized marginal mean difference, comparing clinicians in agencies ± 1 standard deviation from the mean of EBP implementation climate. Cohen [71] suggested *d* could be interpreted as small (0.2), medium (0.5), or large (0.8).

**Fig. 1:**
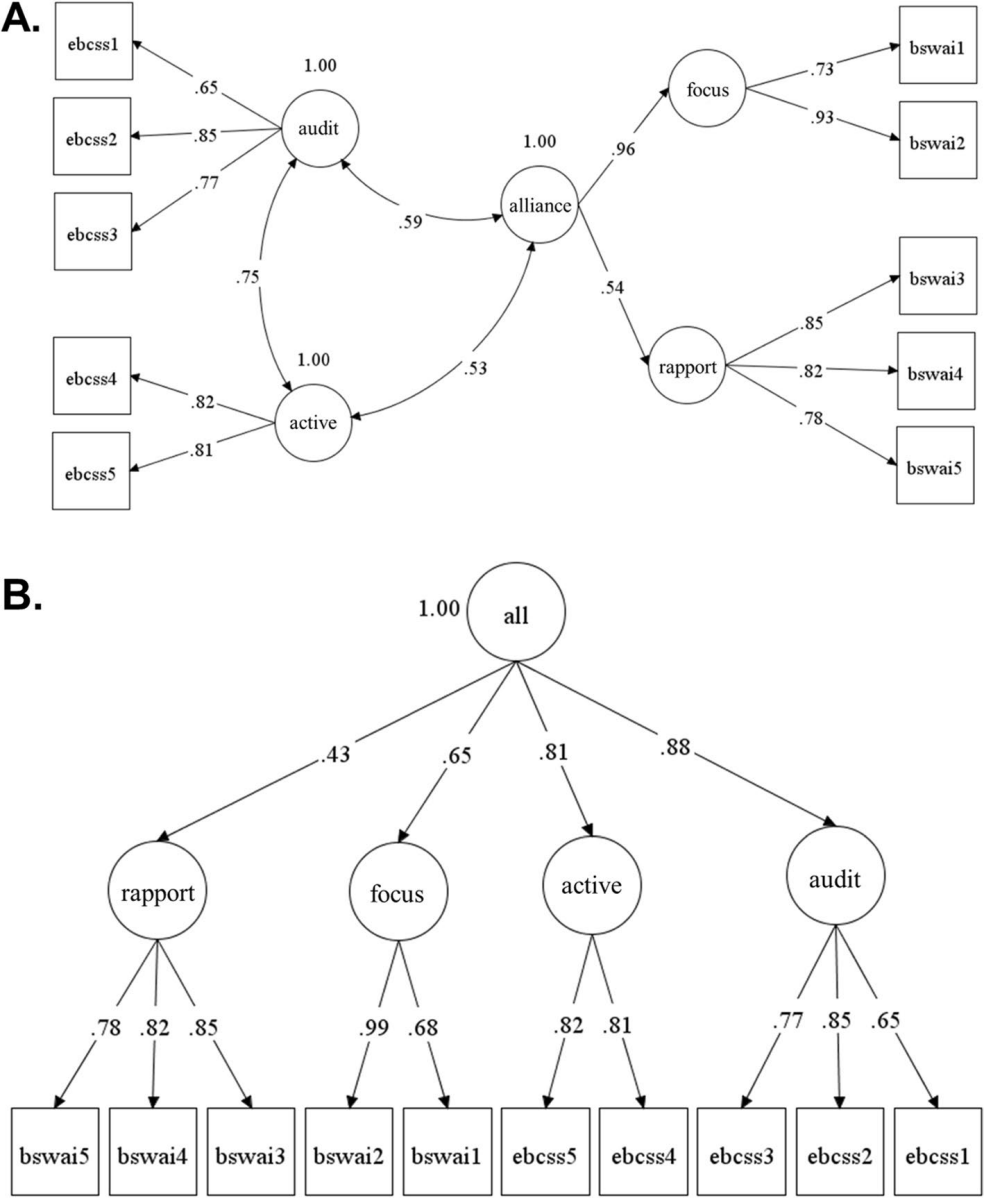
Hypothesized 3-factor model (**A**) and competing 1-factor model (**B**) of EBCSS and BSWAI items. *Note: N* = 181 clinicians. Models estimated using robust maximum likelihood estimation; standardized estimates shown. EBCSS, evidence-based clinical supervision strategies scale; BSWAI-T, brief supervisory working alliance inventory—trainee form; active, active learning subscale of the EBCSS; audit, audit and feedback subscale of the EBCSS; alliance, second-order supervision working alliance factor of the BSWAI-T; focus, client focus subscale of the BSWAI-T; rapport, rapport subscale of the BSWAI-T. Model A: *χ*^2^ = 36.12, *df* = 30, *p* = 0.204; RMSEA = 0.034; CFI = 0.990; SRMR = 0.031. Model B: *χ*^2^ = 55.13, *df* = 31, *p* = 0.005; RMSEA = 0.066; CFI = 0.962; SRMR = 0.067. Results of a Satorra-Bentler scaled chi-square difference test indicated Model A fit significantly better than Model B (S-B Scaled *χ*^2^ Δ = 39.40, *df* = 1, *p* = 0.000)

In aim 2, two CFA models were estimated to assess structural and discriminant validity evidence for scores on the EBCSS. The first model tested the hypothesized factor structure of the EBCSS alongside the hypothesized factor structure of the BSWAI-T (supervisory working alliance) (see Fig. 1A). Based on prior research [55], BSWAI-T items were forced to load onto two first-order latent factors, representing the subscales of rapport and client focus, and these first-order factors were forced to load onto a single second-order factor representing the overall supervisory working alliance (see Fig. 1A). The EBCSS items were forced to load onto their respective factors and these were correlated with each other and with the BSWAI-T second-order factor. Good fit of this model provided evidence supporting [1] the structural validity of scores on the EBCSS and [2] the discriminant validity of scores on the EBCSS relative to the supervisory working alliance.

The second CFA tested a competing hypothesis: scores on the EBCSS and BSWAI-T measure a single, overarching construct (e.g., general likability of the supervisor). In this model, the two EBCSS factors and the two BSWAI-T first-order factors were forced to load onto a single second-order factor (see Fig. 1B). Good fit of this model would undermine the discriminant validity of scores on the EBCSS by suggesting all the scores (BSWAI-T + EBCSS) reflect a single latent construct. A Satorra-Bentler chi-square difference test [72] was used to determine whether the hypothesized 3-factor model fit better than the competing 1-factor model. All models were estimated in Mplus 8 using MLR estimation as described above.

Construct validity evidence for aim 2 was assessed by calculating Pearson correlations between EBCSS scores and other measures of supervision using SPSS 27.

For aim 3, multiple group CFA was used to test the extent to which scores on the EBCSS exhibited measurement invariance across the aim 1 and 2 samples. Measurement invariance is desirable because it suggests item scores assess the same latent construct(s) in the same way across populations, thus supporting generalizability and comparability across populations. This is important because the supervisory actions assessed by the EBCSS are believed to apply across psychosocial EBPs and behavioral health settings.

Following well-established guidelines [73, 74], measurement invariance of scores on the EBCSS was tested by fitting a series of increasingly restrictive multiple group CFA models to data from the samples in aims 1 and 2 and examining the extent to which model fit deteriorated at each step. Specific models provide evidence for different aspects of measurement invariance. The first (least restrictive) model tested configural invariance by imposing the same factor structure in both groups but allowing all parameters to freely vary (i.e., factor loadings, item intercepts, error variances). Support for configural invariance indicates the number of latent constructs, and the alignment of item scores with those constructs is the same across groups [75]. The second (more restrictive) model tested metric invariance. Support for metric invariance indicates the magnitudes of the factor loadings are equal and implies the item scores measure the latent constructs to the same degree in both groups [75]. The third (most restrictive) model tested scalar invariance. Support for scalar invariance indicates “mean differences in the latent constructs capture all mean differences in the shared variance of the items”[74].

The fit of the configural model was evaluated using the model chi-square test and the RMSEA, CFI, and SRMR goodness of fit indices as described above. The extent to which model fit deteriorated when moving from the configural model to subsequent (more restrictive) models was evaluated using the Satorra-Bentler chi-square difference test [72] and by examining change (Δ) in CFI, RMSEA, and SRMR. Measurement invariance was *not* supported if the model chi-square difference test was statistically significant or if there was a change in CFI ≤ −.005, a change in RMSEA ≥ .010, or a change in SRMR ≥ .025 [76]. Given the possibility that full metric or scalar invariance may not be supported, we planned a priori to test for partial metric or scalar invariance as needed following procedures described by Byrne and colleagues [77].

## Results

Table 1 presents the characteristics of the samples for aims 1 and 2. Table 2 presents descriptive statistics and reliability coefficients for the EBCSS items and subscales. Both subscales exhibited adequate score variation; however, as expected, a sizeable proportion of clinicians indicated they had not received any audit and feedback (25%, *N* = 38) or active learning (19%, *N* = 29) during supervision in the last 30 days.

**Table 1:** Characteristics of study participants and supervision

Characteristic	*Aim 1* *N=154*	*Aim 2* *N=181*
**Participants**		
Years of clinical experience (mean ± SD)	6.5 ± 6.2	7.1 ± 32.6
Years tenure in agency (mean ± SD)	3.3 ± 3.8	5.6 ± 5.5
Age (in years) (mean ± SD)	38.9 ± 9.9	42.2 ± 11.9
	***N* (%)**	***N* (%)**
Employment model (%)		
Salaried	66 (42.9)	86 (47.5)
Fee-for-service/contractor	87 (56.5)	95 (52.5)
Race (%)		
Asian	4 (2.6)	5 (2.8)
Black or African American	2 (1.3)	6 (3.3)
American Indian or Alaska Native	0 (0)	2 (1.1)
Native Hawaiian or Other Pacific Islander	2 (1.3)	0 (0)
More than one race	2 (1.3)	NA
White	125 (81.2)	161 (89.0)
Prefer to self-identify	7 (4.5)	4 (2.2)
Prefer not to respond	12 (7.8)	6 (3.3)
Ethnicity		
Identify as Hispanic/Latino	18 (11.7)	8 (4.4)
Do not identify as Hispanic/Latino	134 (87.0)	172 (95.0)
Gender		
Man	26 (16.9)	29 (16.0)
Woman	122 (79.2)	147 (81.2)
Transgender	NA	1 (.6)
Non-binary/non-conforming	NA	3 (1.7)
Prefer to self-identify	5 (3.2)	1 (.6)
Prefer to not respond	NA	2 (1.1)
Education		
Doctoral Degree	6 (3.9)	7 (3.9)
Non-Doctoral Degree	148 (96.1)	174 (96.1)
**Supervision**	**Mean ± SD**	**Mean ± SD**
Total hours per week	2.4 ± 1.7	5.1 ± 4.3
Percent of time on clinical content	59.5 ± 25.0	54.3 ± 28.5
Percent of time on administrative content	29.5 ± 23.4	24.1 ± 24.4
Supervisor availability (1–5 scale)	5.6 ± 1.5	4.5 ± .8

*NA*, not available. No missing responses are included and the percentages do not add up to 100. Aims 1 and 2 did not ask the same question about gender; gender categories were expanded in the table

**Table 2:** Summary statistics and confirmatory factor analysis (CFA) factor loadings for Evidence-based Clinical Supervision Strategies scale items in samples 1 and 2

Item	M	SD	Min-Max	*r*	Standardized factor loading	*α*
Aim 1 (*N* = 154)						
**Clinical performance feedback**	2.17	1.01	1–4.67			0.73
Feedback based on observations.	2.05	1.33	1–5	0.46	0.55	
Feedback based on outcome data.	1.95	1.15	1–5	0.61	0.75	
Feedback based on chart review.	2.52	1.31	1–5	0.59	0.80	
**Active learning strategies**	2.71	1.20	1–5.00			0.76
Role play or rehearsal of a clinical intervention.	2.28	1.30	1–5	0.61	0.75	
Supervisor demonstration of a clinical intervention.	3.14	1.38	1–5	0.61	0.82	
Aim 2 (*N* = 181)						
**Clinical performance feedback**	2.76	1.12	1–5.00			0.79
Feedback based on observations.	2.36	1.37	1–5	0.56	0.65	
Feedback based on outcome data.	2.87	1.32	1–5	0.72	0.85	
Feedback based on chart review.	3.03	1.31	1–5	0.63	0.77	
**Active learning strategies**	2.47	1.19	1–5.00			0.80
Role play or rehearsal of a clinical intervention.	2.15	1.29	1–5	0.66	0.82	
Supervisor demonstration of a clinical intervention.	2.78	1.31	1–5	0.66	0.81	

CFA estimated using robust maximum likelihood estimation. The reported *r* is the corrected item-total correlation

### Reliability

Coefficient alpha for both subscales was acceptable (i.e., *α* > 0.7). Examination of the corrected item-total correlations indicated Item 1 (supervision includes feedback about practice based on supervisor’s in vivo observations or review of audio or video recordings) was not as strongly related to its latent construct as the other items; however, it was retained due to its theoretical importance.

### Structural validity evidence

**Fig. 2:**
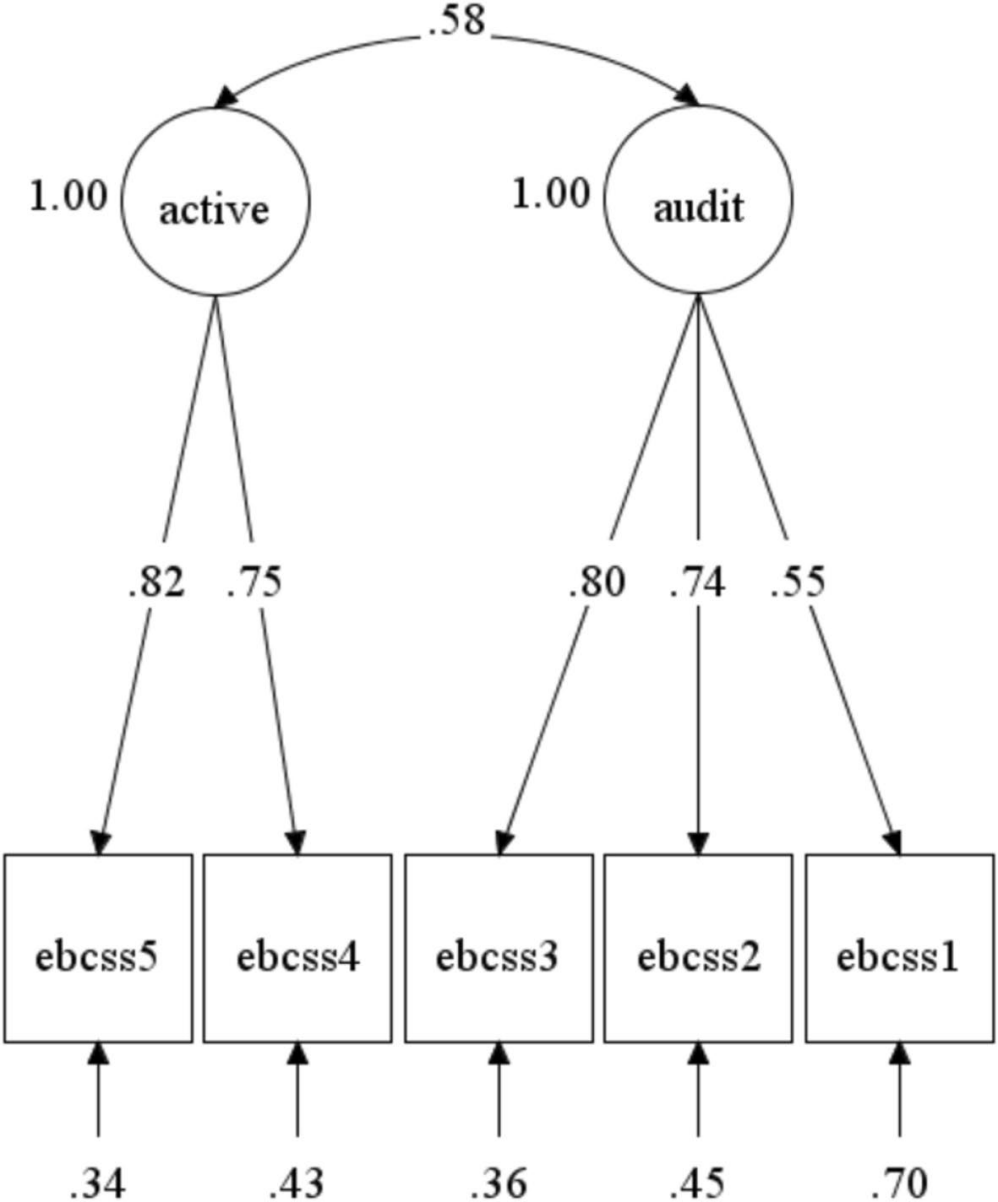
Aim 2 confirmatory factor analysis model

Results of the CFA analyses for aim 1 supported the hypothesized 2-factor structure of scores on the EBCSS. The model was not rejected by the model chi-square test (*χ*^2^ = 5.89, *df* = 4, *p* = 0.208) and all other fit indices were in the good to excellent range (RMSEA = 0.055, CFI = 0.988, SRMR = 0.033). All unstandardized factor loadings were statistically significant at *p* < 0.001 and the standardized factor loadings ranged from 0.55 to 0.82 (see Table 2). The two factors were moderately correlated (*r* = 0.58, *p* < 0.001), providing evidence that the items assessed related but unique supervision experiences (see Fig. 2). The competing 1-factor model, in which all items were forced to load onto a single factor, did not fit the data well and was rejected based on all criteria (*χ*^2^ = 51.40, *df* = 5, *p* = 0.000; RMSEA=0.245, CFI = 0.712, SRMR = 0.077).

### Construct-related validity evidence

**Fig. 3:**
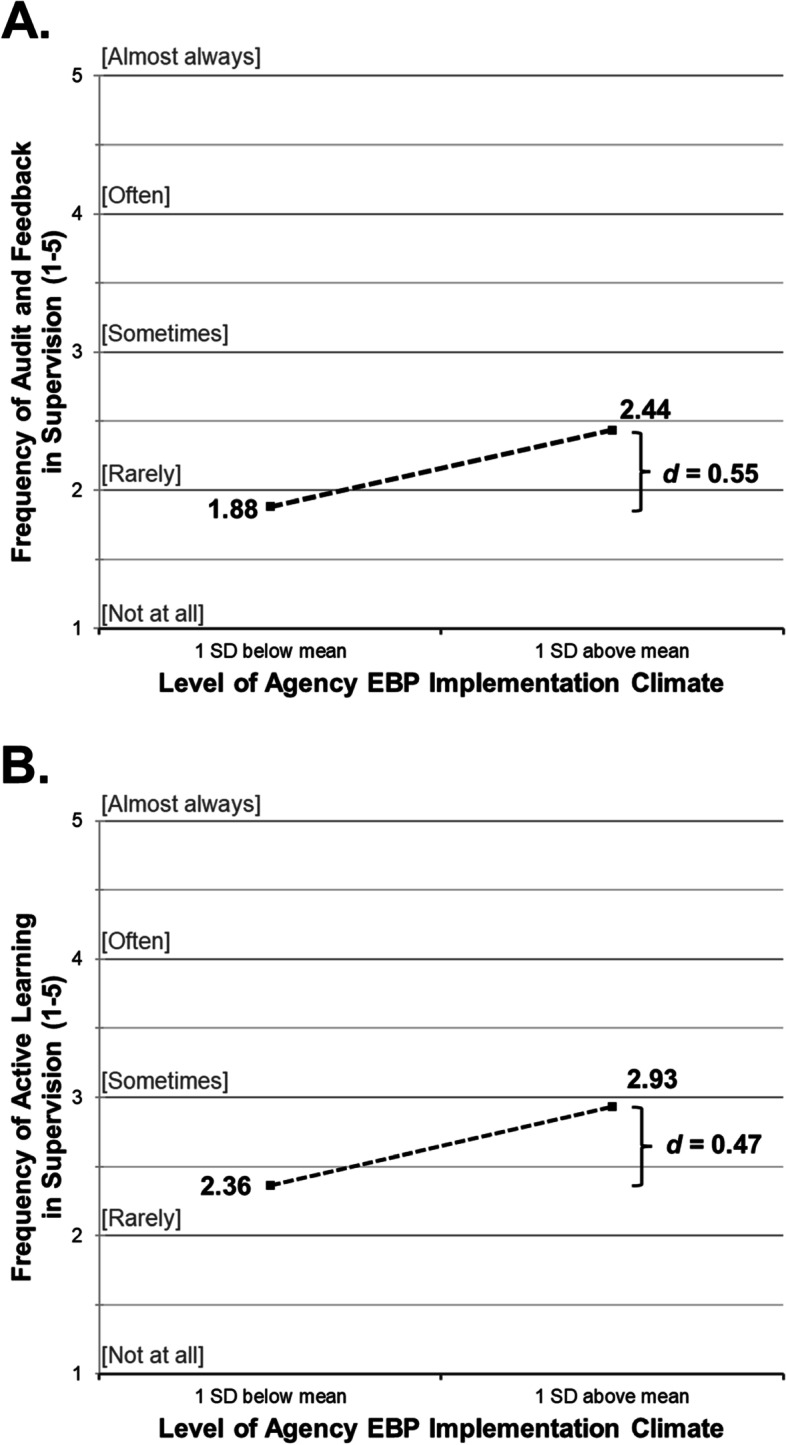
Adjusted mean differences in clinicians’ experience of EBCSS clinical supervision techniques by level of agency climate for EBP implementation. *Note*: *K* = 21 mental health clinics, *N =* 147 clinicians. Adjusted means are estimated using linear 2-level mixed effects regression models with random intercepts; all models control for clinician years of experience and education. EBCSS, Evidence-based Clinical Supervision Strategies scale. ICC[1] for Audit and Feedback = 0.095; ICC[1] for Active Learning = 0.241

Results of the linear mixed-effects regression models for aim 1, which assessed the relationships between agency EBP implementation climate and scores on the EBCSS subscales, are shown in Fig. 3. As expected, higher agency EBP implementation climate predicted greater exposure to audit and feedback in supervision (*B* = 0.28, *p* = 0.010) after controlling for all other variables in the model. This represents a medium effect of *d* = 0.55 (95% CI = 0.13 to 0.96) when comparing the amount of audit and feedback experienced by clinicians in agencies with high (+1 SD) versus low (−1 SD) levels of EBP implementation climate (see Fig. 3A). Clinicians working in agencies with higher levels of EBP implementation climate also reported more exposure to active learning strategies in supervision (*B* = 0.28, *p* = 0.036) representing a medium effect (*d* = 0.47; 95% CI = 0.03 to 0.92) (see Fig. 3B).

### Structural and discriminant validity evidence

Results of the CFA for aim 2, which tested the hypothesized 3-factor model, are presented in Fig. 1A. This model demonstrated excellent fit based on all indices (*χ*^2^ = 36.12, *df* = 30, *p* = 0.204; RMSEA = 0.034; CFI = 0.990; SRMR = 0.031). All unstandardized item factor loadings were statistically significant at *p* < .001 and standardized factor loadings were high (range = 0.65–0.93). As expected, scores on the two EBCSS subscales were correlated (*r* = 0.75, *p* < 0.001) and had moderate but lower magnitude correlations with scores on the supervisory working alliance (*r* = 0.59 and *r* = 0.53, all *p*s < 0.001).

The CFA testing the competing 1-factor model (see Fig. 1B) for aim 2 did not fit the data well and was rejected by the model chi-square test (*χ*^2^ = 55.13, *df* = 31, *p* = 0.005). Furthermore, the Satorra-Bentler chi-square difference test comparing the 1- versus 3-factor models indicated that the 1-factor model fit significantly worse (Δ = 39.40, *df* = 1, *p* < 0.001); consequently, it was rejected. These results offer structural and discriminant validity evidence for scores on the EBCSS.

### Construct validity evidence

Table 3 shows correlations between the EBCSS subscales and the other supervision measures completed as part of aim 2. As expected, small-to-moderate correlations were observed between scores on the EBCSS subscales and the quality of the supervisory exchange and supervisor availability (*r* = .23 to .29). Also consistent with expectations, correlations between the EBCSS subscales and ACT leadership were larger and in the medium range (*r* = .49 and .51, respectively). Finally, inadequate supervision had the anticipated inverse relationships with both EBCSS subscales (see Table 3). These results provide construct validity evidence by showing that scores on the EBCSS are related to, but distinct from, other aspects of supervision in theoretically concordant ways.

**Table 3:** Aim 2 (*N*=181) construct-based validity evidence correlations for EBCSS subscales

	Clinical performance feedback	Active learning strategies
	**r (p)**	**r (p)**
Quality of supervisory exchange	.23 (.002)	.29 (<.001)
ACT leadership	.46 (<.001)	.48 (<.001)
Availability of supervisor	.28 (<.001)	.24 (.001)
Inadequate supervision	−.28(<.001)	−.30 (<.001)

*ACT* Assertive community treatment, *EBCSS* Evidence-based clinical supervision strategies scale

Table 4 presents model fit statistics and change in model fit statistics for the CFA models testing measurement invariance of scores on the EBCSS across the two samples (aim 3). The configural invariance model fit the data well based on all criteria (see Table 4). There was no evidence of significant deterioration in model fit when moving from the configural to the metric invariance model based on the Satorra-Bentler chi-square difference test (Δ = 3.35, *df* = 3, *p* = 0.341) or on changes in CFI, RMSEA, or SRMR. In contrast, results of the Satorra-Bentler chi-square difference test indicated the scalar invariance model fit the data significantly worse than the metric invariance model (Δ = 16.59, *df* = 3, *p* = 0.001) and therefore should be rejected. This conclusion was also supported by deterioration in the values of CFI, RMSEA, and SRMR (see Table 4). Given these results, a partial scalar invariance model was estimated by allowing the intercept for Item 2 to vary freely across groups (“my supervision included feedback about my practice based on data about the people I serve”). As is shown in Table 4, this model exhibited excellent fit based on all criteria (χ^2^ = 18.77, *df* = 13, *p* = 0.130; RMSEA = 0.051; CFI = 0.98; SRMR = 0.042) and there was no evidence of significant deterioration in model fit on any criteria when comparing the partial scalar invariance model to the metric invariance model. Consequently, this model was accepted as final. These results support the configural, metric, and partial scalar invariance of scores on the EBCSS across these two provider samples.

**Table 4:** EBCSS measurement invariance model fit statistics and comparisons

Model	CFI	RMSEA	SRMR	Model *χ*^2^	df	Model *χ*^2^ *p*-value	S-B Scaled *χ*^2^ Δ	df	S-B Scaled *χ*^2^ Δ *p*-value	ΔCFI	ΔRMSEA	ΔSRMR
Configural invariance	0.991	0.054	0.028	11.855	8	0.158						
Metric invariance	0.990	0.048	0.038	15.293	11	0.170	3.350	3	0.341	−0.001	−0.006	0.010
Scalar invariance	0.962	0.082	0.050	29.875	14	0.008	16.589	3	0.001	−0.028	0.034	0.012
Partial scalar invariance^a^	0.986	0.051	0.042	18.770	13	0.130	3.600	2	0.165	−0.004	0.003	0.004

*N* = 335 clinicians (*n* = 154 working in outpatient mental health, *n* = 181 working in assertive community treatment). Models estimated using robust maximum likelihood estimation; *S-B Scaled χ*^*2*^* Δ*, Satorra-Bentler Scaled Chi-Square Difference test

*CFI* Comparative fit index, *EBCSS* Evidence-based Clinical Supervision Strategies scale, *RMSEA* Root mean square error of approximation, *SRMR* Standardized root mean square residual

^a^The intercept for Item 2 (“supervision included feedback about practice based on data about the people I serve”) was allowed to vary freely across groups; all other intercepts and factor loadings constrained equal

## Discussion

The goal of this research was to develop a pragmatic, reliable, and valid measure of clinical supervision as an implementation strategy. Drawing on the literature, clinical supervision was conceptualized as an overarching implementation strategy consisting of two widely applicable, evidence-based techniques: [1] audit and feedback and [2] active learning. The evidence presented here suggests scores on the EBCSS provide a reliable and valid basis for making inferences about the extent to which behavioral health providers experience these techniques as part of their clinical supervision. Across both samples, scores on the EBCSS subscales demonstrated acceptable internal consistency and evidence of structural validity. Construct validity evidence was generated in aim 1 by showing that scores on the EBCSS subscales were higher in agencies with higher levels of EBP implementation climate, an outcome supported by theory and prior research [28]. Aim 2 provided construct validity evidence. Scores on the EBCSS covaried with scores on other measures of the clinical supervision process in anticipated ways, including moderate positive associations with the supervisory alliance and ACT leadership behaviors and negative associations with inadequate supervision behaviors. Aim 3 provided evidence of measurement invariance, suggesting scores on the EBCSS generalize across two settings and populations of behavioral health providers, albeit with some variation in the mean level of data-based feedback provided to the two groups (i.e., partial scalar invariance). Measurement invariance is an important property of scores on implementation measures given the need to evaluate implementation across a range of EBPs and settings.

In addition to its promising psychometric characteristics, the EBCSS aligns well with criteria for pragmatism as described by the PAPERS (Psychometric And Pragmatic Evidence Rating Scale) framework for implementation measures [30]. Specifically, the EBCSS is free (see Additional File 1), brief (5 items), low burden to administer (requires no training), easy to analyze, and understandably written. Because perceptions of pragmatism can vary across stakeholder groups, an important direction for future research is to evaluate the extent to which potential users view the EBCSS as pragmatic across these and other criteria [19, 21].

The EBCSS fills a gap in pragmatic and valid measurement with important applications in research and practice. It can facilitate the identification and optimization of supervision strategies within embedded supervision time in order to promote and sustain provider behavior change. How clinical supervisors use routine supervision time to mediate policy and practice, sell the implementation effort to providers, and diffuse and synthesize information remains less understood [78, 79]. This is particularly important to evaluate across clinical and community-based settings and stages of implementation (i.e., exploration, preparation, implementation, and sustainment). Such research can also unpack the links between a host of organizational context factors (e.g., climate for EBP implementation) and provider implementation behavior [28, 80]. Additionally, including this 5-item measure in clinical and implementation trials will identify effective supervision targets for improved implementation outcomes. Practice applications include evaluating workforce supervision experiences as part of ongoing assessments or quality improvement efforts in order to understand the strengths and gaps in available supports. While rates of these supervision techniques were low, which is consistent with previous literature [12], such gaps highlight the need for growth and improvement to support implementation. Supervision-focused workforce development initiatives could target these techniques to support competent delivery of EBPs. Pursuit of these research and practice applications will help optimize the infrastructure to support widespread and equitable EBP access in routine care.

Further evaluation of the EBCSS is needed. Essential next aims include generation of concurrent criterion-related validity evidence by testing whether scores on this clinician-reported measure correspond with behaviors as rated by trained observers (e.g., via the SPOCS). Studies that generate predictive validity evidence, assess the responsiveness of scores on the EBCSS to changes over time, and further evaluate potential moderating effects of other supervision characteristics and potential expansion to include additional supervision techniques are also needed. Analysis of EBCSS scores using item response theory will further enhance the evaluation of the scores based on the measure.

## Conclusions

This paper advances the conceptualization and measurement of clinical supervision as an implementation strategy. The study presented offers validity evidence indicating scores on the EBCSS form a valid basis for inferences about the extent to which clinicians experience two theoretically grounded, evidence-based clinical supervision techniques that promote the implementation of EBP: audit and feedback and active learning. Findings highlight promising directions for future discovery and provide a tool for stakeholders to optimize the embedded infrastructure of clinical supervision in support of practice improvement.

## Supplementary Information

**Additional file 1:** Evidence-Based Clinical Supervision Strategies Scale (EBCSS).

**Additional file 2:** STROBE checklist.

## Data Availability

NJW and MCB had full access to all the data in the study and take responsibility for the integrity of the data and the accuracy of the data analysis. Requests for access to deidentified data can be sent to Nate Williams at natewilliams@boisestate.edu.

## Abbreviations

BSWAI-T: Br ief Supervisory Working Alliance Inventory—Trainee
CFA: Confirmatory factor analysis
CFI: Comparative fit index
EBCSS: Evidence-Based Clinical Supervision Scale
EBP: Evidence-based practice
MLR: Maximum likelihood estimation
RMSEA: Root mean square error of approximation
SPOCS: Supervision Process Objective Coding System
SRMR: Standardized root mean square residual
