# Supplementary material for: Psychometric evaluation of a pragmatic measure of clinical supervision as an implementation strategy
Source: Implement Sci Commun. 2023 Apr 6;4:39. doi: 10.1186/s43058-023-00419-1 (PMC10080877; doi:10.1186/s43058-023-00419-1)
Supplement: Supplementary file 1 — Additional file 1: Evidence-Based Clinical Supervision Strategies Scale (EBCSS). [file 43058_2023_419_MOESM1_ESM.docx]

**Evidence-Based Clinical Supervision Strategies Scale (EBCSS)**

| Mimi Choy-Brown | Nathaniel J. Williams |
| --- | --- |
| [mchoybro@umn.edu](mailto:mchoybro@umn.edu) | [natewilliams@boisestate.edu](mailto:natewilliams@boisestate.edu) |

This 5-item measure assesses the extent to which supervisees experience evidence-based clinical supervision strategies during their supervision time. The two domains of evidence-based clinical supervision strategies assessed by this scale are: (a) audit and feedback, and (b) active learning during supervision time.

Instructions: Please indicate the extent to which you have experienced each of the following during any of your supervision time in the last 30 days. There are no right or wrong answers to these questions. Please indicate the answer that best represents your experiences during your supervision.

| **Not at all** | **Rarely** | **Sometimes** | **Often** | **Always** |
| --- | --- | --- | --- | --- |
| **1** | **2** | **3** | **4** | **5** |
| **In the last 30 days, …** | | | | |

| 1. … my supervision has included feedback about my practice based on my supervisor’s in vivo observations of my clinical interactions or from review of audio or video recordings. |  | 1 | 2 | 3 | 4 | 5 |
| --- | --- | --- | --- | --- | --- | --- |
| 1. … my supervision has included feedback about my practice based on data about the people I serve (e.g., standardized assessments, outcome measures, satisfaction surveys). |  | 1 | 2 | 3 | 4 | 5 |
| 1. … my supervision has included feedback about my practice based on my supervisor’s review of clinical progress in my charts. |  | 1 | 2 | 3 | 4 | 5 |
| 1. … I have role played or rehearsed a clinical intervention or skill during my supervision. |  | 1 | 2 | 3 | 4 | 5 |
| 1. … my supervisor has demonstrated a clinical intervention or skill during my supervision. |  | 1 | 2 | 3 | 4 | 5 |

**SCORING INSTRUCTIONS**

Items on the EBCSS load onto two latent factors that should be used as separate subscales. The first subscale, “Audit and Feedback,” is calculated by taking the mean of items 1, 2, and 3. The second subscale “Active Learning,” is calculated by taking the mean of items 4 and 5.

Please direct any questions to Mimi Choy-Brown via email at mchoybro@umn.edu.
